# Effect of Megestrol Acetate Combined With Oral Nutrition Supplement in Malnourished Lung Cancer Patients: A Single-Center Prospective Cohort Study

**DOI:** 10.3389/fnut.2021.654194

**Published:** 2021-08-19

**Authors:** Baojun Duan, Yan Zhang, Xi Wang, Yulian Zhang, Yinyin Hou, Jun Bai, Linhua Liu, Yaohua Chen, Rong Zhang, Ronghui Jin, Li He, Yansong Pu

**Affiliations:** ^1^Department of Medical Oncology of Shaanxi Provincial People's Hospital, Xi'an, China; ^2^Department of Cell Biology and Genetics, School of Basic Medical Sciences, Xi'an Jiaotong University, Xi'an, China; ^3^Shaanxi Postdoctoral Innovation Base, Baoji Central Hospital, Baoji, China; ^4^Department of Respiratory and Critical Care of Shaanxi Provincial People's Hospital, Xi'an, China; ^5^Department of Hospital Office of Shaanxi Provincial People's Hospital, Xi'an, China; ^6^Department of Thoracic Surgery of Shaanxi Provincial People's Hospital, Xi'an, China; ^7^Department of Digestive System Diseases of Shaanxi Provincial People's Hospital, Xi'an, China; ^8^Department of Radiology of Shaanxi Provincial People's Hospital, Xi'an, China; ^9^Department of General Surgery of Shaanxi Provincial People's Hospital, Xi'an, China

**Keywords:** lung cancer, nutrition assessment, malnutrition, oral nutrition supplement, megestrol acetate

## Abstract

**Background:** The optimal treatment of cancer-related malnutrition remains unknown. A single-center prospective cohort study was performed to compare the efficacy of megestrol acetate (MA) combined with oral nutrition supplement (ONS) and MA alone for the treatment of lung cancer-related malnutrition.

**Methods:** 76 eligible patients were prospectively enrolled in two arms, Arm 1 patients (*n* = 40, 52.6%) received MA 160 mg/d, and Arm 2 patients (*n* = 36, 47.4%) received MA 160 mg/d combined with ONS 55.8 g/t.i.d, all orally. All patients received anticancer therapy. Treatment duration was 3 months. The primary endpoints were improvements in body mass index (BMI) and Eastern Cooperative Oncology Group (ECOG) score. Secondary endpoints were assessed by appetite, mid-upper arm circumference (MAC), serum pre-albumin levels, and serum albumin levels.

**Results:** Baseline levels were comparable between Arm 1 and Arm 2 patients. Compared with Arm 1, primary endpoints (BMI, *P* = 0.018; ECOG, *P* = 0.022) and secondary endpoints (MAC, *P* = 0.025; serum pre-albumin, *P* = 0.043; and serum albumin, *P* = 0.034) were improved significantly after treatment in Arm 2. While toxicity was negligible and comparable between Arm 1 and Arm 2.

**Conclusion:** MA combined with ONS may be an effective and safe treatment option for lung cancer-related malnutrition patients.

**Clinical Trial Registration:**www.clinicaltrials.gov, identifier ChiCTR2100049007.

## Introduction

Lung cancer is the second most frequently diagnosed cancer and the most common cause of cancer-related deaths worldwide (1). There is a high prevalence of lung cancer-related malnutrition with reported rates ranging from 34.5 to 69%. Lung cancer-related malnutrition is particularly prevalent in patients who have been hospitalized and in those with advanced or metastatic disease (2).

Cancer-related malnutrition, also known as anorexia/cachexia, is an important adverse effect of cancer, which is associated with impaired physical function, diminished tolerance to anticancer therapy, and reduced survival rates (3, 4). Our understanding of malnutrition/cachexia has been greatly improved over the past decades; however, there was no guidelines or standards of care for the treatment of cancer malnutrition/cachexia were universally established until now (4–6). Appetite stimulants such as megestrol acetate (MA), glucocorticoids, non-steroidal anti-inflammatory drugs (NSAIDs), and dronabinol have been extensively studied in patients with cancer-related malnutrition and were reported to stimulate appetite (7). Unfortunately, the use of these appetite stimulants does not often translate to clinically meaningful improvements in lean body mass or functional outcomes (8, 9). Anamorelin has the advantage of stimulating appetite and possibly food intake, as well as promoting anabolism and significant muscle mass gain; however, the availability of drug limits its use (10).

Cancer patients with malnutrition have increased energy needs. In addition to macronutrients, micronutrients such as vitamins and microelements may be beneficial for the treatment of cancer-related malnutrition (11). Therefore, an effective approach is likely to be a combined approach that improves the outcomes of cancer-related malnutrition. As noted in the American Society for Parenteral and Enteral Nutrition (ASPEN) guidelines, enteral nutrition (EN) is preferred over parenteral nutrition (PN) based on the outcomes of hospitalized patients receiving EN therapy. ONS consists of proteins, carbohydrates, fat, vitamins, and minerals, which could be used as the only source of EN or as nutritional supplements (12–14).

The objective of this single-center prospective cohort study was to investigate the efficacy and safety of MA combined with ONS in the treatment of lung cancer-related malnutrition patients.

## Methods

### Study Design

This single-center prospective cohort study compared the efficacy and safety of MA combined with ONS vs. MA in the treatment of lung cancer-related malnutrition. The protocol was approved by the ethics committee of Shaanxi Provincial People's Hospital (20160601). Written informed consent was obtained from all patients. The study was carried out in accordance with Good Clinical Practices and the Helsinki Declaration. The ethical review was carried out by the ethics committee, which ascertained that the study was being conducted in strict compliance with the approved protocol.

The trial was prospectively registered with the Chinese Clinical Trials Registry (ChiCTR2100049007).

### Eligibility and Exclusion Criteria

Patients (18-80 ys of age) with histological confirmed lung cancer, nutrition risk score (NRS) ≥ 3, patient-generated subjective global assessment (PG-SGA) score ≥ B grade, eastern cooperative oncology group (ECOG) score ≤ 3, and a life expectancy ≥ 6 months were eligible. Patients could receive concomitant anti-neoplastic chemotherapy, target or radiation therapy in the palliative medicine setting, or best supportive care. Opioids were allowed for the treatment of cancer pain.

Women of child-bearing age and patients with mechanical obstruction to feeding, drug-induced changes in body weight (corticosteroids for prevention of chemotherapy-induced emesis were allowed), thromboembolism, diabetes mellitus, cardiovascular diseases, such as congestive heart failure (New York Heart Association, NYHA ≥ III), uncontrolled hypertension (systolic pressure > 140 mmHg and diastolic pressure > 90 mmHg), previous myocardial infarction, unstable angina, uncontrolled arrhythmia, previous cerebrovascular accidents, inflammatory bowel diseases, or gastrointestinal ulcers were excluded.

### Intervention

There were no dietary restrictions for those enrolled patients. Arm 1 patients received MA (Xi'an Grand Deten Pharmaceutical Co. Ltd, Xi'an, China) 160 mg/d. Arm 2 patients received MA 160 mg combined with ONS (Abbott Laboratories B. V. Zwolle, Netherlands) 55.8 g/t.i.d (≈750Kcal/d). The treatment duration was 3 months.

### Efficacy Endpoints

The primary endpoints were improvements in body mass index (BMI) and ECOG score. Secondary endpoints were assessed by appetite, mid-upper arm circumference (MAC), serum pre-albumin levels, and serum albumin levels. Appetite was assessed by daily intake, MAC equation used the upper-limb, the mid-way arm circumference measurement of the arm by tape, serum levels of pre-albumin and albumin were measured by immunoturbidimetry assay (Strong Biotechnologies, Beijing, China), and bromocresol green (Fosun Long March, Shanghai, China), respectively. ECOG was evaluated by Zubrod-ECOG-WHO. The endpoints were assessed before treatment and at 3 months after the initiation of treatment. The evaluation of efficacy endpoints was presented in Table 1.

**Table 1:** Evaluation of efficacy endpoints.

	**Effective**	**Stable**	**Ineffective**
Appetite^a^	Increased ≥ 100 g	Increased < 100 g	Decreased > 100 g
BMI	Increased ≥ 1	Increased <1 or decreased <1	Decreased > 1
MAC	Increased ≥ 1 cm	Increased <1 cm or decreased <0.5 cm	Decreased > 0.5 cm
Pre-ALB^b^	Increased	Unchanged	Decreased
ALB^c^	Increased	Unchanged	Decreased
ECOG	Decreased	Unchanged	Increased

*BMI, body mass index; ECOG, Eastern Cooperative Oncology Group performance status; MAC, mid-arm circumference; Pre-ALB, pre-albumin; ALB, albumin*.

a *Appetite was assessed by daily intake*.

b *Unchanged: serum pre-albumin changed by <50 mg/L*.

c *Unchanged: serum albumin changed by <1 g/L*.

### Safety Endpoints

Adverse events including nausea, vomit, diarrhea and constipation were classified according to the National Cancer Institute Common Terminology Criteria for Adverse Events (version 4.0) (15).

### Statistical Analysis

Statistical analysis was performed using GraphPad Prism version 5.0 (GraphPad Software, La Jolla, CA, USA). Quantitative variables were presented as mean ± standard deviation when normally distributed, or as median (interquartile range) if non-normally distributed. Normality was analyzed by using the Kolgomorov-Smirnov test. Qualitative variables were presented as numbers (percentages). The Chi-square test was used to compare categorical variables, and the Student t or Mann Whitney and Wilcoxon tests were used for quantitative variables. A *p*-value of <0.05 was considered significant.

## Results

### Patients

**Figure 1:**
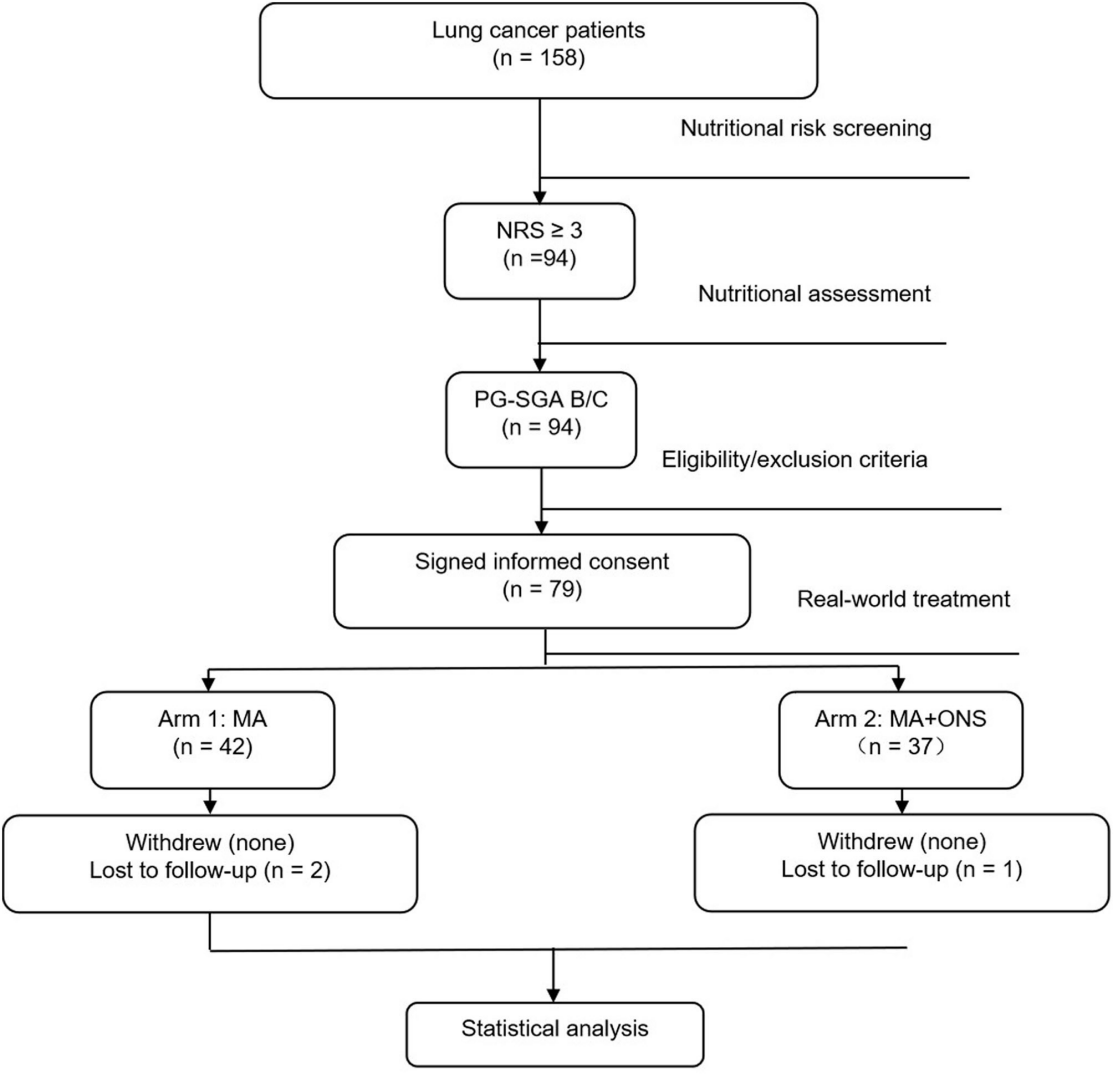
Study Flowchart.

From June 2016 to August 2017, we screened 158 lung cancer patients, of which 94 (59.5%) patients had NRS ≥ 3. NRS was significantly associated with age (*P* = 0.032) and ECOG (*P* = 0.021), which was presented in Table 2. These 94 lung cancer patients had a PG-SGA grade of B/C, and PG-SGA grade was significantly associated with ECOG (*P* = 0.013), which was presented in Table 3. Out of these 94 patients, 79 were enrolled into this study according to the inclusion and exclusion criteria. On the basis of real-world treatments (MA + ONS or MA), these 79 patients were enrolled in Arm 1 and Arm 2, respectively. Three patients (two in Arm 1 and one in Arm 2) were lost to follow-up due to early death as a result of progressive disease. Therefore, 76 lung cancer patients were evaluated ultimately (Figure 1). The patients enrolled in each arm were comparable at baseline on the basis of the most common stratification factors (Table 4).

**Table 2:** Relationship between clinical features and NRS.

**Clinical features**	**NRS ≥ 3** **No. (%)**	**NRS < 3** **No. (%)**	***P*-value**
Gender			0.348^*^
Male	57 (60.6)	34 (53.1)	
Female	37 (39.4)	30 (46.9)	
Age			0.032^*^
≥60	63 (67.0)	32 (50.0)	
<60	31 (33.0)	32 (50.0)	
ECOG			0.021^*^
>2	34(36.2)	35 (54.7)	
>2	60 (63.8)	29(45.3)	

*NRS, nutritional risk screening*.

* *Chi-square test was used*.

*Bold type refers to statistically significant differences*.

*The meaning of the underline value is statistically significant differences*.

**Table 3:** Relationship between clinical Features and PG-SGA.

**Clinical features**	**PG-SGA**	***P*-value**	
	**Grade B** **No. (%)**	**Grade C** **No. (%)**	
Gender			0.673^*^
Male	16 (61.5)	45 (66.2)	
Female	10 (38.5)	23 (33.8)	
Age			0.067^*^
≥60	14 (53.8)	50 (73.5)	
<60	12 (46.2)	18 (26.5)	
ECOG			0.013^*^
>2	12 (46.2)	14 (20.6)	
>2	14 (53.8)	54 (79.4)	

*PG-SGA, patient-generated subjective global assessment*.

* *Chi-square test is used*.

*Bold type refers to statistically significant differences*.

*The meaning of the underline value is statistically significant differences*.

**Table 4:** Baseline characteristics of patients.

**Clinicopathological parameters**	**Arm 1** **No. (%)40**	**Arm 2** **No. (%)36**	***P*-value**
Gender			0.790^*^
Male	21 (52.5)	20 (55.6)	
Female	19 (47.5)	16 (44.4)	
Age(ys)			0.431^*^
≥60	22 (55.0)	23 (63.9)	
<60	18 (45.0)	13 (36.1)	
Weight(Kg)	55.2 ± 9.1	52.6 ± 10.6	0.516^#^
Height(m)	1.64 ± 0.08	1.66 ± 0.12	0.772^#^
BMI(Kg/m^2^)	20.3 ± 3.5	19.2 ± 4.1	0.635^#^
Histology			0.852^*^
SCLC	5 (12.5)	4 (11.1)	
NSCLC	35 (87.5)	32 (88.9)	
Stage			0.792^*^
I-III	10 (25.0)	6 (16.7)	
IV	30 (75.0)	30 (83.3)	
ECOG			0.415^*^
<2	28 (70.0)	22 (61.1)	
≥2	12 (30.0)	14 (38.9)	
ALB(g/L)			0.498^*^
<35	22 (55.0)	17 (47.2)	
≥35	18 (45.0)	19 (52.8)	
Concomitant therapy			0.971^*^
Chemotherapy	15 (37.5)	14 (38.9)	
Radiotherapy	2(5.0)	3 (8.3)	
Target therapy	6(15.0)	5 (13.9)	
Immune therapy	5 (12.5)	4 (11.1)	
Palliative therapy	8 (20.0)	8 (22.2)	
Surgery	4 (10.0)	2 (5.6)	

* *Chi-square test is used*.

# *Student t-tests is used*.

### Efficacy Endpoints

Compared to Arm 1, the primary endpoint (BMI and ECOG) improved in Arm 2 patients significantly (*P* = 0.018 and *P* = 0.022, respectively). Moreover, there were significant improvements in secondary endpoints (MAC, serum pre-albumin and serum albumin) in Arm 2 patients than that of Arm 1 (*P* = 0.025, *P* = 0.043, and *P* = 0.034, respectively). The absolute values of serum pre-albumin and serum albumin in Arm1 at baseline and after the 3-month trial is 169.19 ± 68.68mg/L, 171.50 ± 59.79mg/L and 32.20 ± 5.76g/L, 33.70 ± 5.68g/L, and the absolute values of serum pre-albumin and serum albumin in Arm2 at baseline and after the 3-month trial is 133.19 ± 75.42mg/L, 182.89 ± 66.96mg/L and 34.14 ± 6.57g/L, 36.96 ± 4.99g/L, respectively. These data was presented in Table 5.

**Table 5:** Changes in efficacy endpoints.

**Endpoints**	**Arm 1** **No. (%)**	**Arm 2** **No. (%)**	***P*-value**
BMI			0.018^*^
Increased	17 (42.5)	25 (69.4)	
Decreased	23 (57.5)	11 (30.6)	
ECOG			0.022^*^
Increased	25 (62.5)	13 (36.1)	
Decreased	15 (37.5)	23(73.9)	
Appetite			0.281^*^
Increased	23 (57.5)	25 (80.6)	
Decreased	17 (42.5)	11(19.4)	
MAC			0.025^*^
Increased	20 (50.0)	27 (75.0)	
Decreased	20 (50.0)	9 (25.0)	
Pre-ALB			0.043^*^
Increased	25 (62.5)	30 (83.3)	
Decreased	15 (47.5)	6 (16.7)	
ALB			0.034^*^
Increased	22 (55.0)	28 (77.8)	
Decreased	18 (45.0)	8 (22.2)	

* *Chi-square test is used*.

*Bold type refers to statistically significant differences*.

*The meaning of the underline value is statistically significant differences*.

### Safety Assessment

Toxicities induced by the study treatment were negligible and comparable between Arm1 and Arm 2 (Table 6). Grade 3 nausea led to the withdrawal of ONS for 4 days. Overall, patient compliance was adequate.

**Table 6:** Toxic effects.

**Toxicity**	**Arm 1** **(No.)**	**Arm 2** **(No.)**	***P*-value**
Nausea			0.809^*^
Grade 1/2	2	3	
Grade 3/4	1	1	
Vomit			0.361^*^
Grade 1/2	1	2	
Grade 3/4	0	1	
Diarrhea			0.576^*^
Grade 1/2	1	3	
Grade 3/4	0	1	
Constipation			-
Grade 1/2	0	0	
Grade 3/4	0	0	
Thromboembolism			0.439^*^
Grade 1/2	3	2	
Grade 3/4	0	1	

* *Chi-square test is used. - Means cannot statistics analysis*.

## Discussion

Considering that adequate nutrition has important effects on health outcomes, both nutritional risk screening and nutritional assessment in cancer patients are critical. Nutrition risk score 2002 (NRS2002) and PG-SGA are recommended for nutritional risk screening and nutritional assessment in cancer patients (9–12). Dietetic assessment and intervention in lung cancer (DAIL) trial revealed that PG-SGA identified 78% of patients required specialist nutritional advice, with 52% patients having a critical need for dietetic input and symptom management (16). Higher malnutrition risk and elevated inflammatory status in patients with lung cancer were associated with poor overall survival independently (17).

In our study, 94 (59.5%) out of 158 lung cancer patients had nutritional risks (NRS ≥ 3). Moreover, nutritional risks were significantly associated with age and ECOG. Out of these 94 patients, 26 (27.7%) were PG-SGA grade B, and 68 (72.3%) were PG-SGA grade C. PG-SGA was significantly associated with ECOG. Abbass et al. Showed that compared to low nutrition risk, patients at moderate to high risk had poor ECOG, elevated frailty index, elevated modified Glasgow prognostic score (17); However, further research is required to determine whether a low nutritional score leads to a low ECOG or a low ECOG contributes to a low nutritional score.

MA was approved for cancer-associated malnutrition in Europe, consequently, the most commonly prescribed drug for this condition. MA stimulates appetite by affecting metabolism and proinflammatory cytokine synthesis (15, 18). Many articles had reported that MA was more effective than other drugs, such as dronabinol and fluoxymesterone (19, 20). Combined medical therapy using MA and fish oil, dronabinol, or NSAIDs resulted in no changes in weight or appetite compared to MA alone (21, 22). Beta-hydroxy beta-methyl butyrate/arginine/glutamine(HMB/Arg/Gln) may work together to decrease muscle damage from reactive oxygen species and pro-inflammatory cytokine; however, recent data demonstrated that HMB/Arg/Gln supplementation couldn't improve the loss of lean body mass in patients with advanced lung cancer (23).

Compared to PN, EN causes fewer complications, is cheaper, and equally effective. EN carries a low risk of serious complications, reduces bacterial translocation from the intestinal tract to the systemic circulation, derating levels of circulating inflammatory cytokines, helps to restore normal intestinal function, and cut down infectious complications and overall costs of care (24).

Because of appetite stimulants such as MA and the others (including dronabinol, fluoxymesterone and NSAIDs et al.) alone could not translate appetite increase to clinically meaningful improvements in lean body mass or functional outcomes (8, 9). We tried to compare the efficacy and safety of two management approaches (appetite stimulants combined EN) in lung cancer patients with malnutrition. Compared with MA alone, MA combined with ONS significantly improved MAC, BMI, ECOG and serum levels of pre-albumin and albumin. This outcome may be associated with that MA + ONS improved the quality and quantity of nutrients intake on the basis of stimulating appetite. Treatment-related toxicity was negligible and comparable between arms, and patient compliance was adequate.

This study suffered from a few limitations that deserve to be underlined. Firstly, our study was limited by its single-center design. Secondly, we were unable to provide alternative methods to measure anthropometric parameters, e.g., dual-energy-x-ray absorptiometry or body plethysmography; However, it should be noted that BMI and MAC were still considered reliable and inexpensive anthropometric measurement (25).

## Conclusion

Our study revealed that MA combined with ONS may be an effective and safe treatment option for lung cancer-related malnutrition.

## Data Availability Statement

The original contributions presented in the study are included in the article/Supplementary Material, further inquiries can be directed to the corresponding author/s.

## Ethics Statement

The studies involving human participants were reviewed and approved by Medical Ethics Committee of Shaanxi Provincial People's Hospital. The patients/participants provided their written informed consent to participate in this study.

## Author Contributions

BD, YP, LH, YaZ, LL, YH, RJ, and RZ were involved in the design of the study, the conception of the case report form, patients' recruitment, data collection, and drafting of the manuscript. YuZ and JB supervised the study design. YaZ and BD contributed to data interpretation and manuscript preparation. YP, YC, and JB contributed to statistical analysis and interpretation of data. All authors contributed to the critical revising and the final approval of the manuscript.

## Conflict of Interest

The authors declare that the research was conducted in the absence of any commercial or financial relationships that could be construed as a potential conflict of interest.

## Publisher's Note

All claims expressed in this article are solely those of the authors and do not necessarily represent those of their affiliated organizations, or those of the publisher, the editors and the reviewers. Any product that may be evaluated in this article, or claim that may be made by its manufacturer, is not guaranteed or endorsed by the publisher.

## Abbreviations

ONS: Oral Nutrition Supplement
MA: Megestrol Acetate
BMI: Body Mass Index
ECOG: Eastern Cooperative Oncology Group
MAC: Mid-upper Arm Circumference
NSAIDs: Non-Steroidal Anti-Inflammatory Drugs
ASPEN: American Society for Parenteral and Enteral Nutrition
PN: Parenteral Nutrition
NRS: Nutrition Risk Score
PG-SGA: Patient-Generated Subjective Global Assessment
NYHA: New York Heart Association
NRS2002: Nutrition Risk Score 2002.
