# Supplementary material for: Effect of Megestrol Acetate Combined With Oral Nutrition Supplement in Malnourished Lung Cancer Patients: A Single-Center Prospective Cohort Study
Source: Front Nutr. 2021 Aug 19;8:654194. doi: 10.3389/fnut.2021.654194 (PMC8416678; doi:10.3389/fnut.2021.654194)
Supplement: Supplementary file 1 [file Table_1.pdf]

Arm1				Arm2			
Pre-ALB		ALB		Pre-ALB		ALB	
Baseline	3-months	Baseline	3-months	Baseline	3-months	Baseline	3-months
188.5	210.1	22.2	25.8	194.2	340.1	22.5	34
92.1	101.7	25.9	36.2	204.5	266.3	23.7	28.5
170	80.2	26.4	31.8	274.1	213.4	23.9	33.2
162.2	117.4	29	28.3	131.6	187.2	24.3	39
144.7	240.8	30.2	38.5	75.1	189.0	24.4	30
188	69.2	30.5	34.6	93.2	151.8	24.6	34.1
54.7	100.6	32.2	31.8	114.9	60.6	26.5	28
274.1	216.5	32.4	39.6	119.6	206.1	28.7	34.6
289.7	176.8	33.9	30.1	168	90.1	29.2	27
185.8	190.6	34	25.2	159.8	61.4	30.3	33.4
248.6	260.8	34.9	36.1	15.3	182.3	30.7	33
177.1	249.5	25.2	29.4	265.6	210.5	30.7	34.9
143.1	172.1	25.5	38.7	45.7	190.2	31.5	30
162.1	169.4	26.1	35.5	110.9	185.6	31.8	40.5
153.1	209.5	26.5	34.9	102.4	214.9	33.5	32.1
217.1	289.1	26.6	33.6	192.1	280.1	34.2	35.6
160.8	48.2	26.7	19.6	118.4	182.4	34.8	37
133.3	160.7	27	33.2	73.4	19	35.1	41.5
202.2	215.8	27.2	36.5	293.7	226.3	35.2	37
115.7	165.1	27.5	31.9	245.3	299	35.5	37.3
270.6	221.8	27.6	25.2	77.8	150.3	35.7	40.7
49.7	202.5	28.6	21	30.6	114.8	35.8	36.9
270.4	210.3	29	25.6	70	136.5	36	39
89.4	152.4	29.2	37.6	140.5	214.5	36.8	38.2
205.3	165.2	29.9	23.5	143.5	197.3	37.9	39.6
179.2	199.6	40	36.4	14.6	166	38	35.6
238.5	70.1	30.1	33.8	112.2	203.5	38.5	43.1
189.5	227.4	34.4	35.7	68.5	163.2	38.7	40
150.7	120.6	35.2	34.6	249.6	187.9	38.7	36
195.3	248.2	36.1	35.3	211.2	261.7	39.3	42.5
23.5	205.2	36.5	36.6	148.7	204.8	41.7	39
112.8	169.6	36.7	39	37.6	119.4	41.7	43
199.8	187.2	37.5	36.2	135.6	190	42.7	39.4
27	104.9	38.6	39.3	39.2	160.2	43.3	46.8
164.9	55.8	39.2	35.9	184.3	110.3	45.3	47.2
153.5	119.3	40.1	37.2	133.1	247.4	47.7	43
202.4	210.8	40.5	40.1				
100	192.5	41.4	43.7				
318.2	162.2	43.2	40.5				
164	190.2	44.2	39.4				
