# Supplementary material for: Effect of Megestrol Acetate Combined With Oral Nutrition Supplement in Malnourished Lung Cancer Patients: A Single-Center Prospective Cohort Study
Source: Front Nutr. 2021 Aug 19;8:654194. doi: 10.3389/fnut.2021.654194 (PMC8416678; doi:10.3389/fnut.2021.654194)
Supplement: Supplementary file 2 [file Table_2.pdf]

The components of the ONS (TP, ENSURE®, Abbott Labs)

**Main(per 100g)**							
Energy	450kcal	Protein	15.9g	Fat	15.9g	Linoleic acid	8.7g
Carbohydrate	60.7g	Water	5g	Biotin	150mg	Choline	136mg
Pantothenic acid	5.0mg						
**Vitamin(per 100g)**							
Vit. A	1170IU	Vit. B1	0.72ug	Vit. B2	0.80ug	Vit. B6	1.0ug
Vit. B12	3.1ug	Vit. C	68ug	Vit. D3	95IU	Vit. E	10.7IU
Vit. K1	18ug	Folic acid	200ug	Niacin	10.0ug		

**Minerals(per 100g)**									
K	670mg	Na	360mg	Cl	610mg	Ca	230mg	P	230mg
Fe	4.37mg	Mn	1.2mg	I	34ug	Cr	20ug	Mg	90mg
Zn	5.4mg	Cu	0.52mg	Se	20ug	Mo	38ug		

Vit.: vitamin.
